# The Impact of the Postpartum “Doing-the-Month” Practice on Human Milk Microbiota: A Pilot Study in Taiwan

**DOI:** 10.3390/microorganisms8091283

**Published:** 2020-08-22

**Authors:** Po-Wen Chen, Yu-Hsien Kuo, Yi-Ling Lin

**Affiliations:** 1 Department of Veterinary Medicine, National Chung Hsing University, Xingda Road, Taichung 40249, Taiwan; 2 Department of Nursing, St. Mary’s Junior College of Medicine, Nursing and Management, Yilan 26647, Taiwan; hsien6184@smc.edu.tw; 3 Department of Obstetrics Central, St. Mary’s Hospital, Luodong 26546, Taiwan; dr2752@smh.org.tw

**Keywords:** doing the month, human milk, microbiota, postpartum practice

## Abstract

Human milk microorganisms could benefit the healthy development of the immune system in infants. In Asia, the practice of “doing-the-month” indicates a month-long period of postpartum recuperation for new mothers. This is composed of cultural practices, traditional beliefs, behavioral, dietary, and herbal therapies. In this pilot study, we evaluated the effect of “doing-the-month” on the human milk microbiota using a molecular approach. We collected two “doing-the-month” milk groups from randomly recruited mothers who had completed their “doing-the-month” program in either postpartum care center A (milk-PCA, *n* = 14) or postpartum care center B (milk-PCB, *n* = 27) for 20 to 30 days. As for the control group, milk samples were selected from postpartum mothers (milk-H, *n* = 46), who did not conduct the “doing-the-month” program. We found that the “doing-the-month” milk samples were associated with more diverse and unique milk microbiota and that these samples were also linked with more abundant *Lactobacillus* (milk-PCB) and prevalent *Bifidobacteria* (milk-PCA and milk-PCB). In addition, the milk samples from “doing-the-month” mothers could be enriched with more Archaea bacterial members, but the “non-doing-the-month” milk samples were enriched with more common skin-, oral-, and environmental-related bacterial members. This study highlights the impact maternal practices may have on the milk microbiome. More research is needed to investigate the effects this may have on infant immune health.

## 1. Introduction

Human milk is a complex fluid and is the optimal postnatal nutrition for infants. It contains essential nutrients such as proteins, vitamins, minerals, fats, and carbohydrates, and a broad range of immune cells and bioactive components [1,2]. Breastfeeding has therefore been shown to promote health in infants. It is associated with a reduction in diseases such as diarrhea [3], necrotizing enterocolitis [4,5], respiratory infection [3,6], enterovirus infection [7], and allergies, although the evidence for this is conflicting in some cases [8].

Mounting evidence supports the presence of human milk containing its own microbiota, which could have health-related implications, particularly in maintaining the mammary gland health of the mother [9,10,11] and establishing the intestinal microbiome of the infant and contributing to nutrient metabolism, immunity against infection, and the maturation and regulation of the immune system [12]. Several factors are currently known to be involved in the establishment of the intestinal microbiome in infants, and a connection between the human milk microbiota and the intestinal microbiota of the infant has been reported [12]. For example, there is a correlation between mother–infant pairs in breast milk and infant feces in the relative abundance of probiotics such as *Bifidobacteria adolescentis*, *B. bifidum*, *B. breve*, and *Lactobacillus plantarum* [13,14,15]. Moreover, the transfer of *Staphylococcus* spp. [16], *Lactobacillus* spp. [16,17] and *Bifidobacterium* spp. [16] from human milk to the infant intestine has been reported as well. A recent study also proposed that breast milk could act as a reservoir of *Lactobacilli* for the infant’s gut microbiota, which would then benefit infant health [18]. Several studies are attempting to determine the factors that could influence the milk microbiota, with a recent review summarizing these factors. These factors include the geographic location, gestational age, delivery mode, biological sex, parity, intrapartum antibiotics, lactation stage, diet, body mass index (BMI), composition of breast milk, human immunodeficiency virus (HIV) infection status, and collection/feeding method, which could impact the breast milk microbiota composition. This review also highlights that several previous studies were limited and their findings were contradictory [19]. We hypothesize that the contradiction in the findings of previous studies could be due to the above factors, which have different effects on the milk microbiota and are challenging to account for in a study. It is still worthwhile to understand the factors that could drive the variation in the milk microbiota since the milk microbiota is likely transferred directly to breast-fed infants. This hypothesis is supported by the observation that *Lactobacillus* spp., administered as a probiotic to mothers, can be detected in their breast milk [10,11,20]. However, the complex nature of the milk microbiota should be noted, particularly in light of a report which showed retrograde inoculation, whereby the microbiota in the infant oral cavity impacted the milk microbiota [21].

The Chinese custom of postpartum confinement, “doing-the-month” or “zuoyuezi”, is widespread across Chinese-speaking societies in several Asian and Western countries. This complex practice is a 20–30 day ritual involving physical and social prescriptions, traditional beliefs, and dietary and herbal therapy. For example, this encourages postpartum mothers to rest completely (many stay in bed) throughout the postnatal period, and new mothers should avoid sexual intercourse, going outside or going to the temple, and becoming cold. Moreover, postpartum mothers should avoid eating functionally “cold” foods, such as salad, bamboo shoots, leafy green vegetables, turnips, Chinese cabbage, cold drinks, and ice products. In contrast, postpartum mothers are encouraged to eat functionally “hot” or “warm” foods, such as food cooked with ginger and sesame oil, rice wine, kidney, liver, chicken, and eggs. Furthermore, Chinese herbal medicines, such as Sheng-Hua-Tang and Szu-Wu-Tang, are frequently used by postpartum women during pregnancy. These warm foods and herbal medicines are believed to improve wound healing, prevent infection and chronic illness, and increase lactation for breast feeding [22,23,24,25,26]. This ritual can be practiced at home or at postpartum care centers. At present, quite a few postpartum mothers are interested in practicing this ritual, where the 20–30 day program, inclusive of behavioral, dietary, and herbal therapies, has been integrated with the nursing routines and care provisions [24,25]. Albeit with some differences [27,28], several studies indicate that this practice can provide both physiological and psychological benefits to postpartum mothers [23,25,29,30]. Collectively, the practice of “doing-the-month” could promote maternal postpartum recovery and lactation, and we propose that this practice may also influence the milk microbiota, although it has not been studied yet. In this pilot study, we compared the milk microbial composition between “doing-the-month” and “non-doing-the-month” milk samples. Initially, we selected two postpartum care centers that provide the typical program of “doing-the-month” and determined the milk microbiota using a molecular approach. This pilot study conducted in Taiwan is the first to assess the influence of “doing-the-month” on the human milk microbiota.

## 2. Materials and Methods

### 2.1. Milk Sample and Practice of “Doing-the-Month” in Postpartum Care Centers

The ethical approval for this study and all study protocols was approved by the Ethics Committee of the Institutional Review Board (IRB) of Saint Mary’s Hospital, Lundong (Project code: IRB105013 and IRB107010). All participants gave their informed consent for inclusion before they participated in the study, and written informed consent was obtained from all the participants. Two postpartum care centers were selected that provide a typical “doing-the-month” service but with a variety of dietary and herbal therapies. Briefly, milk samples were collected from postpartum mothers who had completed the practice of “doing-the-month” (postpartum care) for 20 to 30 days in postpartum care center A (milk-PCA, *n* = 14) or B (milk-PCB, *n* = 27). The control milk samples were harvested from postpartum mothers in hospital who did not undergo the “doing the month” (milk-H, *n* = 46) program. The relative abundance of all the bacterial genera in each milk sample was determined using 16S rRNA gene amplicon sequencing, as described in the methods section. The milk samples were collected and stored following previously reported protocols [31,32].

### 2.2. DNA Extraction

The total bacterial DNA in milk was directly extracted according to the protocol reported in our previous study using the QIAsymphony^®^ Virus/Bacteria Mini Kit (Qiagen, Düsseldorf, Germany) [32]. A 1 mL milk sample was centrifuged at 5000× *g* for 30 min, and the supernatant was discarded. The bacterial pellet was collected and re-suspended in 300 μL of an enzyme solution (20 mg/mL lysozyme or 200 μg/mL lysostaphin in a buffer containing 20 mM Tris-HCl pH 8.0, 2 mM EDTA, and 1.2% Triton X-100) at 37 °C for at least 30 min. The total bacterial DNA was extracted according to the manufacturer’s instructions, and the obtained DNA samples were further subjected to a microbial diversity analysis.

### 2.3. Milk Microbiota Analysis

The microbial composition of the milk samples was determined using Illumina MiSeq sequencing. The obtained bacterial DNA samples from milk were subjected to an initial 30-cycle PCR using AccuPrime Hifi Polymerase (Invitrogen, Carlsbad, CA, USA), as described in our previous study [33]. The cycling conditions were as follows: initial denaturation at 94 °C for 2 min, followed by 30 cycles at 94 °C for 20 s, 56 °C for 30 s, 68 °C for 60 s, and final storage at 4 °C. Amplicons of the variable regions V3 to V4 of the 16S rRNA gene were amplified with primers 341F (5′-CCTACGGGNGGCWGCAG-3′) and 805R (5′-GACTACHVGGGTATCTAATCC-3′) (Illumina, Carlsbad, CA, USA). Libraries were purified using AMPure XP beads (LABPLAN; Naas, Ireland) according to the Illumina 16S sequencing library protocol, and the libraries were tested for purity and quantity using a Nanodrop 1000 spectrophotometer. The barcoded amplicon libraries were then combined in equal concentrations into a single pool according to their Nanodrop-based quantification. The size of each amplicon was assessed with an Agilent DNA 1000 Kit (Agilent Technologies Ireland Ltd., Dublin, Ireland) on an Agilent 2100 Bioanalyzer (Agilent Technologies Ireland Ltd., Dublin, Ireland). The pooled amplicon libraries were sequenced using the MiSeq system, producing 2 × 300 bp paired-end reads. For the microbial diversity analysis, the steps to obtain the final reads were performed according to the manufacturer’s protocol. The Illumina-generated FASTQ files (fastq) and quality files were acquired as raw and mapped sequence data. These data were further analyzed using the latest version of the bioinformatics software packages Mothur v.1.33.3 and QIIME v1.80 for the 16S rDNA analysis (e.g., the selection of operational taxonomic units (OTUs) and taxonomic assignment) by means of the Greengenes 16S rRNA database (gg_13_8). Here, the pairwise distances between the aligned DNA sequences for all effective reads were calculated with a cutoff of 0.03, then clustered into OTUs using the average neighbor algorithm with a hard cutoff of 0.03. The OTUs were finally classified by taxonomic assignment.

### 2.4. Statistical Analysis

To compare the microbiota structure among the milk groups, alpha diversity indices (Shannon, Simpson reciprocal diversity, and Chao1 index) were established to compare the microbial richness among the milk groups. Moreover, beta diversity indices, such as principal component analysis (PCA) and principal coordinate analysis (PCoA; weighted and unweighted UniFrac distances for PCoA), were used to explore the clustering of subjects [33,34,35]. The Wilcoxon rank-sum (Mann–Whitney 2-sample) test was performed to evaluate the alpha diversity indices among the milk groups using the SPSS 20 statistical software (SPSS Inc., Chicago, IL, USA). Furthermore, the Linear Discriminant Analysis Effect Size (LEfSe) algorithm, including the bar and Cladogram graphs, was also applied to identify and evaluate the more abundant taxa in one group compared to the other groups [36]. Venn diagrams (Venny 2.1) were used to depict the unique and shared sets of taxa lists between the milk groups.

## 3. Results

### 3.1. Information for Milk Groups

The maternal demographics were similar and are shown in Table 1. Here, the two postpartum care centers provided dietary therapies often complemented with herbal therapies (supported by the theory of Traditional Chinese Medicine). However, the frequency and the contents of the dietary therapies differed between the two postpartum care centers. Briefly, Postpartum Care Center A provided 3 to 5 meals per day, and their meals were often complemented with a few characteristic herbal therapies. These included Shi-Quan-Da-Bu-Tang (ten-significant tonic decoction), Ba-zhen-tang (eight-treasure decoction), Eucommia leaf tea, herbal medicine recipes (business confidentiality), and herbal tea (promotes lactation). Finally, postpartum mothers who had completed their practice of “doing-the-month” for at least 20 days (20 to 30 days) in postpartum care center A were randomly recruited, and milk samples were collected and harvested from the mothers. These milk samples (or milk group) were designated as milk-PCA (*n* = 14). In contrast, postpartum care center B provided 5 meals per day, which were often complemented with fewer herbal items, such as Sheng-hau-tang, Siwu-Tang, Eucommia leaf tea, and special herbal medicine recipes. The postpartum mothers who had completed their practice of “doing-the-month” in this institute for 20 to 30 days were also recruited randomly, and the milk samples were collected and harvested from them. These milk samples were designated as the milk-PCB group (*n* = 27). The postpartum care centers all served a higher frequency of seafood for the nourishment of the postpartum mothers. It should be also be noted that the herbal therapy is often complementary or an alternative for postpartum mothers, and thus each mother would receive a unique combination of therapies as prescribed by the doctors in the postpartum care centers. All the recruited mothers in this study had rested and received care in the postpartum care centers for 20 to 30 days, and there were 10-, 20-, or 30-days programs for the mothers to select from in the Postpartum Care Centers.

We also collected milk samples from postpartum mothers who did not undergo the practice of “doing the month” as controls, and these samples were designated as milk-H (*n* = 46). The total number of samples obtained was 14, 27, and 46 milk samples for the milk-PCA, milk-PCB, and milk-H groups, respectively.

### 3.2. Prevalence of Lactobacillus or Bifidobacteria in the Three Milk Groups

There has been some contradiction in the abundance and distribution of probiotics (*Lactobacilli* and *Bifidobacteria*) in human milk samples in previous reports [37,38]. Thus, we initially focused on the distribution of milk probiotics between “doing-the-month” and “non-doing-the-month” milk groups. To detect the presence of all the *Bifidobacteria* and *Lactobacillus* bacteria in milk samples, all the detected molecular signals were included here. As shown in Table 2, the *Bifidobacteria* bacteria abundance in milk-H, milk-PCA, and milk-PCB groups was 32.6%, 62.9%, and 57.1%, respectively. The *Lactobacilli* in milk-H, milk-PCA, and milk-PCB groups was 86.9%, 100%, and 92.8%, respectively. These data reveal that *Lactobacilli* was more prevalent than *Bifidobacteria* in all the milk groups. The *Bifidobacteria* abundance was also two-fold higher in the “doing-the-month” milk than in “non-doing-the-month”.

### 3.3. Proportion of Major *and* Minor Bacterial Genera in the Milk Groups

**Figure 1:**
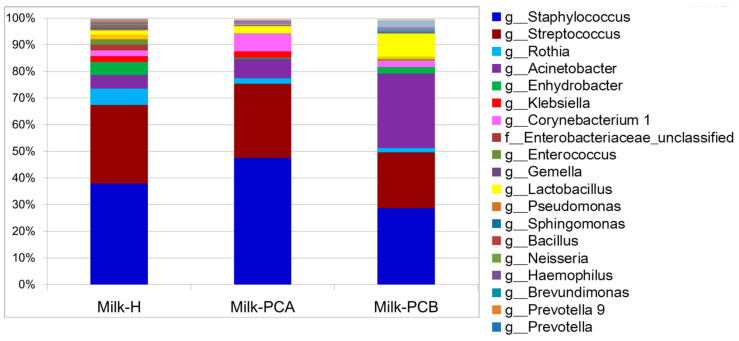
Proportions of major bacterial genus between two “doing-the-month” and one “non-doing-the-month” milk groups. Milk samples were collected from postpartum mothers who had completed the practice of “doing-the-month” (postpartum caring) for 20 to 30 days in postpartum care center A (milk-PCA, *n* = 14) or B (milk-PCB, *n* = 27). The control milk samples were harvested from the postpartum mothers in hospital who did not conduct the “doing-the-month” (milk-H, *n* = 46). The relative abundance of all the bacterial genera in each milk sample was determined using 16S rRNA gene amplicon sequencing, and the results were plotted according to the different milk groups.

We then compared the proportion of all the major bacterial genera between the milk groups (Figure 1). The most common bacterial genera in the “non-doing-the-month” milk group (milk-H) were *Staphylococcus* (37.9%), *Streptococcus* (29.5%), *Rothia* (6.2%), *Acinetobacter* (5%), *Enhydrobacter* (4.9%), *Klebsiella* (2.2%), *Corynebacterium* (2.2%), *Enterobacteriaceae* (2.2%), *Enterococcus* (2%), *Gemella* (1.7%), and *Lactobacillus* (1.6%).

The major bacterial genera in milk-PCA were *Staphylococcus* (47%), *Streptococcus* (27.5%), *Acinetobacter* (7.1%), *Corynebacterium* (6.6%), *Lactobacillus* (2.6%), *Klebsiella* (2.2%), *Rothia* (2.1%), and *Methylobacterium* (1.2%). In the “non-doing-the-month” milk group, the major or common bacterial genera in milk-PCB were *Staphylococcus* (28.5%), *Acinetobacter* (27.8%), *Streptococcus* (20.8%), *Lactobacillus* (8.6%), *Enhydrobacter* (2.4%), *Corynebacterium* (2.4%), *Sphingobium* (2.4%), and *Rothia* (1.6%).

The relative abundance of *Bifidobacteria* was not recognized (Figure 1), as it constituted 0.07%, 0.03%, and < 0.01% of the milk-H, milk-PCA, and milk-PCB groups, respectively. Moreover, *Lactobacillus* constituted approximately 1.6%, 2.6%, and 8.6% of the milk-H, milk-PCA, and milk-PCB groups, respectively. These findings concur with the results shown in Table 2. Collectively, *Bifidobacteria* was less prevalent than *Lactobacilli* (Table 2, Figure 1) in all the milk samples, regardless of group. The two “doing-the-month” milk groups contained a higher proportion of *Lactobacilli* than the “non-doing-the-month” milk groups, and milk-PCB harbored *Lactobacilli* at amounts as high as 8.6%.

In summary, the three milk groups displayed different profiles for major bacterial populations. For example, milk-H contained a higher abundance of *Rothia* and *Enhydrobacter*, while milk-A contained more *Corynebacterium*, and milk-PCB contained more *Acinetobacter* and *Lactobacillus*. Apparently, *Lactobacilli* was more abundant in the two “doing-the-month” milk groups compared to the “non-doing-the-month” milk group. We further compared the relative proportion of *Lactobacilli* in each milk sample between the three milk groups. Several previous studies have indicated that human milk could harbor about 2–3% *Lactobacilli* and *Bifidobacteria* (relative abundance) [14,39,40,41]. However, most milk samples in our study were colonized with less than 2% relative abundance of *Lactobacilli*, regardless of the milk group. Thus, we compared the relative abundance of *Lactobacilli*, which was higher than 3% in a particular milk sample between the three milk groups. Here, *Lactobacillus* was at 4.9%, 35%, and 75.3% relative abundance in three milk-PCA samples, and 4.7%, 14.4%, 19.4%, 3.5%, and 24% relative abundance in five milk-PCB samples. *Lactobacillus* accounted for 18.2% and 49.9% relative abundance in two milk-H samples. In other words, there were 4.3% (2/46), 21.4% (3/14), and 18.5% (5/27) milk samples that had been colonized with a higher *Lactobacillus* population (relative abundance) in the milk-H, milk-PCA, and milk-PCB groups, respectively.

The data above shows a different tendency towards the proportion and prevalence of specific bacterial members between the three milk groups. Two additional analysis methods were used to compare the characteristics and specific bacteria between the milk groups.

### 3.4. Linear Discriminant Analysis Effect Size (LEfSe) Analysis to Identify Differences in Abundant Microbial Taxa between the Three Milk Groups

**Figure 2:**
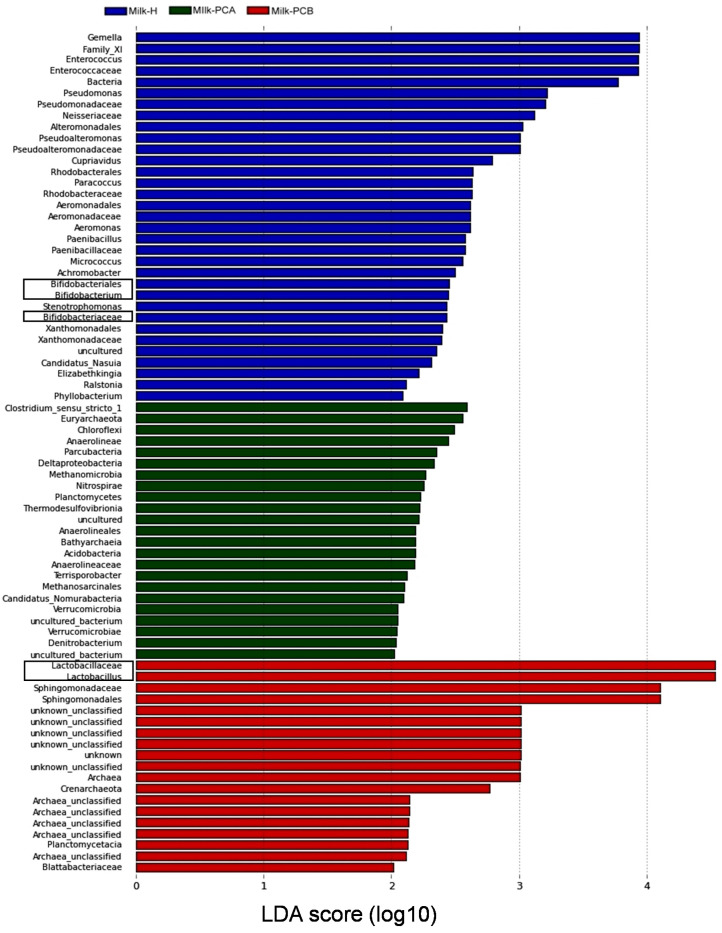
Different abundant microbial taxa between two “doing-the-month” and one “non-doing-the-month” milk groups revealed using a linear discriminant analysis effect size (LEfSe) analysis. The keys for milk-H, milk-PCA, and milk-PCB are described in Table 1. Briefly, milk-H (*n* = 46): “non-doing-the-month” milk group; milk-PCA (*n* = 14) and milk-PCB (*n* = 27): “doing-the-month” milk group. Bar graph showing bacteria that are more abundant in milk-H (blue color), milk-PCA (green color), and milk-PCB (red color) ranked and identified using the linear discriminant analysis (LDA) score. The probiotic bacteria (lactobacilli and bifidobacteria) were indicated in the rectangles within the figure.

To identify and compare the significantly microbial differences between the “doing-the-month” and “non-doing-the-month” milk groups, a linear discriminant analysis effect size (LefSE) method was employed here [36]. In Figure 2, LEfSe identified 33, 20, and 8 groups of bacterial genera enriched in the milk-H, milk-PCA, and milk-PCB groups, respectively (LDA score > 2). This indicated a total of 61 bacterial groups that were differentially abundant between the milk groups, implying that the three milk groups are indeed colonized with a variety of characteristic bacterial members. It should be noted that some identified bacterial members in milk-PCB are actually unknown and unclassified, and these were not counted here.

The LEfSe identified 33 bacterial genera enriched in the milk-H group, and the top 10 enriched bacterial members in this milk group were *Gemella*, Family_X1, *Enterococcaceae*, *Pseudomonas*, *Pseudomonadaceae*, *Neisseriaceae*, *Alteromonadales*, *Pseudoalteromonas*, *Pseudoalteromonadaceae*, and *Cupriavidus*. Furthermore, the LEfse also identified *Bifidobactericeae* and *Bifidobacterium* as enriched in this group as well.

The LEfSe identified 20 bacterial membranes enriched in milk-PCA, and the top 10 enriched bacterial members (from top to bottom) were *Clostridium* sensu stricto, *Euryarchaeota*, *Chloroflexi*, *Anaerolineae*, *Parcubacteria*, *Deltaproteobacteria*, *Methanomicrobia*, *Nitrospirae*, *Planctomycetes*, and *Thermodesulfovibrionia*. In contrast, LEfSe identified eight bacterial membranes enriched in milk-PCB, indicating that the presence of *Lactobacillus*, *Sphhinogomonadaceae*, *Sphingomonadales*, *Archaea*, *Crenarchaeota*, *Planctomycetacia*, and *Blattabacteriaceae* was enriched in the milk-PCB group.

### 3.5. Cladogram Analysis to Identify the Greatest Differences in Microbial Taxa between the Milk Groups

**Figure 3:**
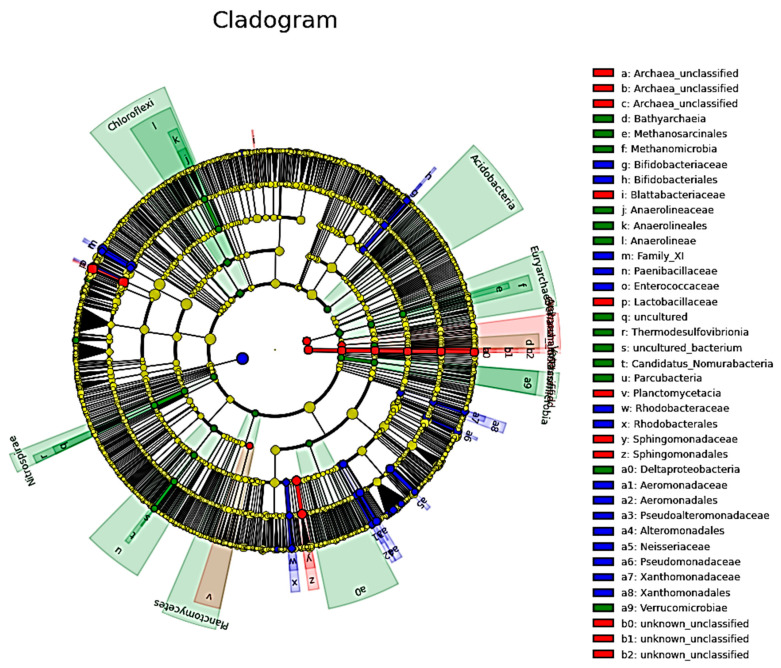
Different abundant microbial taxa between two “doing-the-month” and one “non-doing-the-month” milk groups revealed using Cladogram, which analyzed using the Linear discriminant analysis effect size (LEfSe). The keys for milk-H, milk-PCA, and milk-PCB are described in Table 1. Briefly, milk-H (*n* = 46): “non-doing-the-month” milk group; milk-PCA (*n* = 14) and milk-PCB (*n* = 27): “doing-the-month” milk group. Graphs and bacterial members that are more enriched in milk-H (blue color), milk-PCA (green color), and milk-PCB (red color) are shown.

We also conducted a cladogram analysis to identify the greatest differences in taxa between the milk groups (Figure 3). This analysis is based on the LEfSe analysis but uses a graphical representation of evolutionary history. As shown in Figure 3, this analysis identified 15 bacterial groups in the milk-H group, including *Bifidobacteriaceae*, *Bifidobacteriales*, Family_XI (Clostridiales Family), *Paenibacillaceae*, *Enterococcaceae*, *Aeromonadaceae*, *Aeromonadales*, *Pseudoalteromonadaceae*, Alteromonadales, *Neisseriaceae*, *Pseudomonadaceae*, and *Xanthomonadales*. Moreover, this analysis also identified 12 bacterial groups enriched in milk-PCA, including *Bathyarchaeia*, *Methanosarcinales*, *Methanomicrobia*, *Anaerolineaceae*, Anaerolineae, *Thermodesulfovibrionia*, *Candidatus*_*Nomurabacteria*, *Deltaproteobacteria*, and *Verrucomicrobiae*. Finally, this analysis identified five bacterial groups in milk-PCB, including *Archaea*, *Blattabacteriaceae*, *Lactobacillaceae*, *Planctomycetaci*, *Sphingomonadaceae*, and *Sphingomonadales*. In comparison to the results shown in Figure 2, the results obtained from the Cladogram analysis concur with the findings of the LEfSe analysis.

### 3.6. Analysis of the Similarity *and* Diversity of Milk Microbial Communities between “Doing-the-Month” *and* “Non-Doing-the-Month” Groups

By using the prevalence, relative abundance, LEfSe, and cladogram analysis above, our data support that “doing-the-month” and “non-doing-the-month” milk groups could harbor similar characteristics or a common microbiota composition. We then conducted an alpha diversity analysis to further evaluate the similarity and diversity of the microbial communities between the milk groups, and also conducted a beta diversity analysis to evaluate the similarity and diversity of the microbial communities between the milk groups and each milk sample.

**Figure 4:**
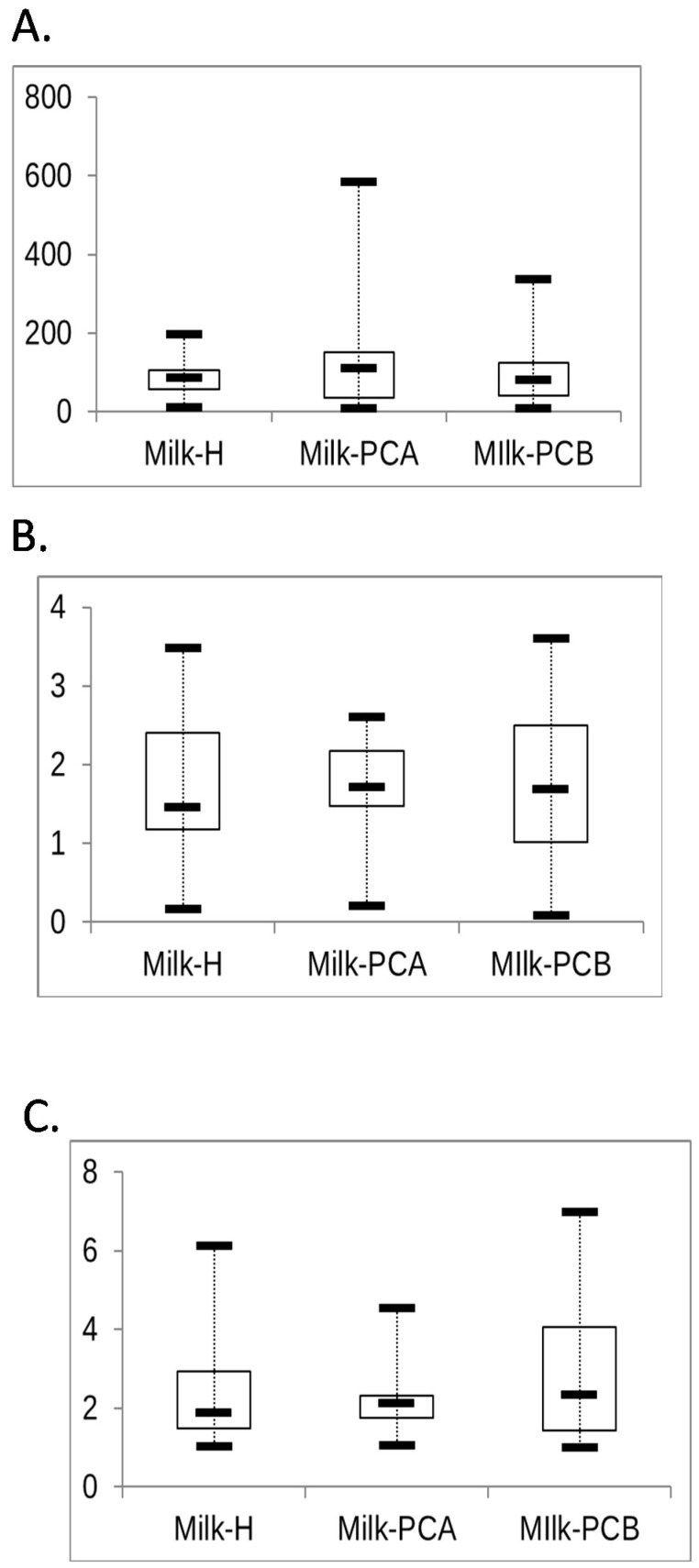
Alpha diversity between two “doing-the-month” and one “non-doing-the-month” milk groups. The keys for milk-H, milk-PCA, and milk-PCB are described in Table 1. Briefly, milk-H (*n* = 46): “non-doing-the-month” milk group; milk-PCA (*n* = 14) and milk-PCB (*n* = 27): “doing-the-month” milk group. The Chao 1 index (**A**), Shannon index (**B**), and Simpson reciprocal index (**C**) were computed for three milk groups. The box depicts the interquartile range between the first and third quartiles (25th and 75th percentiles, respectively), and the line inside the box indicates the median.

In Figure 4, three alpha analyses, Shannon, Chao1, and the Simpson Reciprocal Index, were selected to evaluate the alpha diversity between the milk groups (Figure 4). In Figure 4A, the Chao1 analysis estimated the diversity from the abundance data (observed species/importance of rare OTUs), and obtained the index for each milk sample and depicted it together using the Box-and-Whisker plot. The findings show that (Figure 4A) the median species were 84, 109, and 79 for milk-H, milk-PCA, and milk-PCB, respectively, and the indices of the median did not show statistical differences, per the Wilcoxon rank-sum test (*p* > 0.05). These data indicate that the practice of “doing-the-month” would not affect the “median numbers” of bacterial species between the three milk groups. However, an apparently wider interquartile range for the Chao1 indexes can be recognized for the milk-PCA and milk-PCB group (doing-the-month milk group) when compared to that for the milk-H group. For instance, the interquartile ranges (between the 25th and 75th percentiles) are 56–105, 35–151, and 41–124 species in milk-H, milk-PCA, and milk-PCB, respectively. Furthermore, the maximum number of recognized bacterial species was approximately 200, 600, and 350 for the milk-H, milk-PCA, and milk-PCB groups, respectively. Therefore, in comparison to the interquartile range and maximum number for the recognized bacterial species of “non-doing-the-month” milk group (milk-H), a large portion of the milk samples in the “doing-the-month” milk groups tended to contain more bacterial species. We performed another two alpha diversity analyses to determine the microbial diversity between the milk groups. Simpson’s diversity index was used to quantify the microbial diversity of a single milk sample, while considering both the species richness (the number of species recorded in the sample) and the relative species abundances. Notably, Simpson’s index is more related to the relative abundances. Moreover, the Shannon index was also selected to evaluate the microbial diversity of a single milk sample, but it focused more on species richness. In Figure 4B,C, box-and-whisker plots were used to depict the results from the same milk groups. Here, the median values of the Shannon and Simpson Reciprocal Index for the three milk groups were similar, and did not show statistical difference as per the Wilcoxon rank-sum test (*p* > 0.05). The interquartile ranges between the 25th and 75th percentiles for the Shannon index in milk-H and milk-PCB were also similar, and milk-PCA displayed a narrow range of indices. Accordingly, the findings using the Simpson reciprocal index were similar to those of the Shannon index. Notably, we could recognize a wider range for interquartile ranges (between the 25th and 75th percentiles) and also a wider range for the lowest and highest indexes. These data implied that a large portion of milk samples displayed a unique microbial diversity profile, regardless of the milk group. To further compare the overall differences between milk samples from the different milk groups, a beta diversity analysis was conducted as described below.

**Figure 5:**
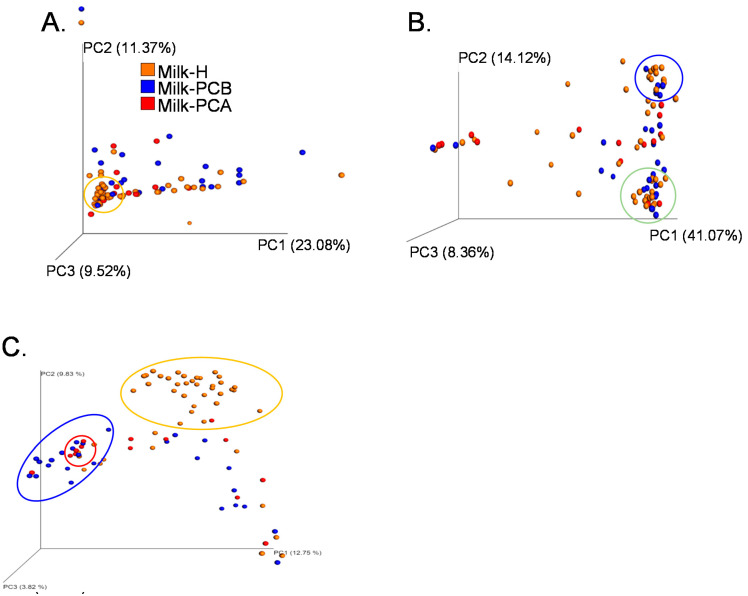
Beta diversity analysis of the milk microbiota between two “doing-the-month” and one “non-doing-the-month” milk groups. Principal component analysis (**A**), principal coordinate analysis based on the weighted UniFrac metric distance (**B**) and unweighted UniFrac metric distance (**C**) of three milk groups are shown. The keys for milk-H, milk-PCA, and milk-PCB are described in Table 1. Briefly, milk-H (*n* = 46): “non-doing-the-month” milk group; milk-PCA (*n* = 14) and milk-PCB (*n* = 27): “doing-the-month” milk group. The orange circle in (**A**) indicates the dimensional area for the clustered milk samples. The green or blue circle in (**B**) indicates the two-dimensional area for the clustered milk samples. The orange, blue, or red circle in (**C**) indicates the three-dimensional area for the relatively aggregated milk samples.

In Figure 5, beta diversity analyses, such as principal component analysis (PCA) and principal coordinates analysis (PCoA), were conducted to evaluate the microbial diversity between the milk samples and milk groups. The above analyses were distance-based multivariate analyses that combined the location and dispersion effects. For example, both variables, the abundance of bacterial genera and the pattern of bacteria in each milk sample, were evaluated, and when two samples were found to have similar patterns of bacterial genera they were placed in the same dimensional space.

As shown in Figure 5A, a PCA analysis revealed that some milk samples from the three milk groups were clustered together, as marked by the orange circle within the figure. For example, 27 milk-H samples were clustered in the same dimensional space, and these were clustered together with another 9 milk-PCB and 3 milk-PCA samples. Except for the relatively aggregated milk samples, the other milk samples of the “doing-the-month” milk groups (milk-PCA and milk-PCB) were distributed discreetly in the dimensional space, indicating that these milk samples contained a unique or different bacterial profile. In summary, in the milk-H group, approximately 59% of the samples (27/46) shared similar bacterial profiles, and 45% (27+9+3/87) of all milk samples shared similar bacterial profiles. On the other hand, in the two “doing the month” groups, approximately 29% (9+3/41) of the milk samples displayed a similar microbiota. A large portion (about 59%) of “non-doing-the-month” milk samples displayed a similar bacterial composition, but most (about 71%) “doing-the-month” milk samples displayed different bacterial profiles. To confirm these findings, we performed the weighted (quantitative) variants of UniFrac distance analysis to compare the milk microbiota between each milk sample or milk group (Figure 5B).

In Figure 5B, two clustered areas of milk samples can be observed. For example, about 10 milk-H samples aggregated closely with 5 milk-PCB samples (marked by the blue circle within the figure). Another 16 milk-H samples clustered closely with 9 milk-PCB samples and 2 milk-PCA samples (marked by the green circle within the figure). In other words, approximately 21.7% (10/46) and 34.7% (16/46) of the samples in milk-H shared a similar bacterial profile through the weighted variants of the UniFrac distance analysis. This indicated that 56.4% of the milk-H samples displayed similar but different bacterial profiles (two clusters). These results were similar to the findings obtained using PCA analysis, as they revealed that approximately 59% of the milk-H samples share a similar bacterial profile, as described above. As indicated above, about 14 milk-PCB samples (5 samples in the blue circle and another 9 samples in green circle within Figure 5B) were located closely in two-dimensional space, indicating approximately 18% (5/27) or 33% (9/27) of the milk-PCB samples shared two kinds of similar bacterial profiles. As for the milk-PCA group, approximately 14% of the samples (2/14) clustered and shared a similar bacterial profile. Therefore, the findings obtained using the weighted variants of UniFrac distance analysis are similar to the results obtained using the PCA analysis. Collectively (Figure 5A,B), about 67% to 86% of the “doing-the-month” milk samples tended to share a different bacterial profile, but only about 41% to 43% of the “non-doing-the-month” milk samples tended to share a different bacterial profile.

We also employed the unweighted UniFrac metric distance to compare the microbiota composition between the above milk groups (Figure 5c). Only two samples of milk-PCA, three samples of milk-PCB, and two samples of milk-H clustered in the same dimensional space (shown by the rec circle in Figure 5C), but the other milk samples were distributed separately in the dimensional location, indicating that most milk samples did not share a similar bacterial composition in the analysis of the unweighted UniFrac metric distance. It should be indicated that the unweighted PCoA analysis only considers the presence or absence of a bacterial community [34,35] and, as expected, most samples shared no similar bacterial compositions, while only taking the presence or absence of specific bacterial profiles into account. It should be noted that although most milk-H samples did not cluster together, approximately 85% (23 milk samples) of the milk-H samples were located in the near dimensional space, as marked by the orange circle in Figure 5C, and these samples were also distributed away from most samples of the milk-PCA and milk-PCB groups. Furthermore, approximately 55% (15 milk samples) of the milk-PCB samples were also distributed in the near dimensional space (marked in the blue circle). Therefore, the findings of the unweighted UniFrac analysis support that, although most milk samples display a unique bacterial profile (only considering presence or absence), a milk-group-relevant distribution of the bacterial community could be found, and this was especially obvious in the milk-H group. In other words, the unweighted UniFrac analysis also confirmed that approximately 85% of the milk samples in the milk-H group and 55% of the milk samples in milk-PCB could share a relatively similar bacterial profile. These findings concur with the results obtained using the PCA and weighted UniFrac metric distance analysis (Figure 5A,B).

The data above revealed the diversity of microbial composition of each milk sample between three milk groups and that a large portion of the “doing-the-month” milk samples shared a unique bacterial profile, but a relatively small portion of the “non-doing-the-month” milk samples had different bacterial profiles. We further compared the whole bacterial constitution in the three milk samples as described below.

### 3.7. Common and Different Bacterial Genera and Species between the Three Milk Groups

**Figure 6:**
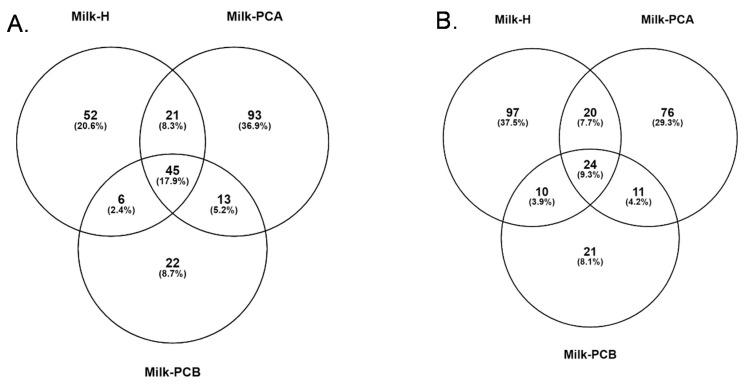
Common and different bacterial genera and species between the two “doing-the-month” and one “non-doing-the-month” milk groups. Venn diagrams showing the number of bacterial genera (**A**) or species (**B**) shared between or unique to the three milk groups, excluding bacterial genera or species present at an abundance of < 1%. The keys for milk-H, milk-PCA, and milk-PCB are described in Table 1. Briefly, milk-H: “non-doing-the-month” milk group; milk-PCA and milk-PCB: “doing-the-month” milk group.

In Figure 6, the whole bacterial constitutions, both unique and shared, were compared using a Venn analysis. In Figure 6A, the milk-H and milk-PCA samples shared 26.2% identical bacterial genera, and milk-H and milk-PCB shared 20.3% of the same bacterial genera. Moreover, milk-PCA and milk-PCB shared 23.1% identical bacterial species. Furthermore, the unique bacterial genera were 20.6%, 36.9%, and 8.7% in the milk-H, milk-PCA, and milk-PCB samples, respectively. In Figure 6B, the milk-H and milk-PCA samples shared 17% identical bacterial species, and milk-H and milk-PCB shared 13.2% identical bacterial species. Milk-PCA and milk-PCB shared 13.2% identical bacterial species. The unique bacterial species were observed to be at 37.5%, 29.3%, and 8.1% in the milk-H, Milk-PCA, and milk-PCB samples, respectively. Notably, some OTUs from the MiSeq approach which had not been recognized as unique bacterial genera or species were not evaluated in the Venn analysis. In summary, these results partially confirmed the observation of different specific bacterial members in the three milk groups using LefSE and cladogram analysis, as described above (Figure 2 and Figure 3).

## 4. Discussion

This pilot study conducted in Taiwan is the first study, to our knowledge, which assessed the overall influence of “doing-the-month” on the human milk microbiota. Our investigation emphasizes the impact maternal practices can have on the microbiome, and this may be an important contributor to infant health, but more work is needed.

Some previous studies have reported a diverse range of milk *Lactobacilli* and *Bifidobacteria* in human milk [14,32,39,40,41,42]. Therefore, we were interested in whether the practice of “doing-the-month” may influence the distribution of probiotics (*Lactobacilli* or *Bifidobacteria*) in human milk. Intriguingly, statistical analyses (LEfSe and Cladogram analysis; Figure 2, Figure 3) have identified and recognized that *Lactobacillus*-related bacteria are enriched in the milk-PCB group (one “doing-the-month” milk group). Furthermore, the incidence and relative abundance of *Lactobacilli* in milk-PCB are 92.8% and 8.6%, respectively. Moreover, approximately 18.5% (5/27) of the milk samples of this milk group were found to be colonized with relatively higher *Lactobacilli* populations (more than 3% relative abundance in each milk). In contrast, in the milk-H milk group (“non-doing-the-month” milk), the incidence and relative abundance of *Lactobacillus* were 86.9% and 1.6%, respectively. Only 4.3% (2/46) of the milk samples in milk-H were colonized with a much higher *Lactobacilli* population. As for another “doing-the-month” milk group (milk-PCA), the relative abundance of *Lactobacillus* in milk-PCA was 2.6%. Although this value is similar to previous reports (2–3%), the incidence of *Lactobacilli* in this milk group is 100%. Furthermore, approximately 21.4% (3/14) of the milk samples of the milk-PCA group were colonized with relatively higher *Lactobacilli* populations (more than 3% relative abundance in each milk). As mentioned above, only 4.3% of the milk samples in milk-H are observed to be colonized with relatively higher *Lactobacilli* populations. All these findings support that the milk samples from mothers who completed the “doing-the-month” could be more abundant in *Lactobacillus*-related bacteria. Thus, we proposed that the relatively higher population of *Lactobacilli* in some “doing-the-month” milk could act as a reservoir of *Lactobacilli* for the infant’s gut microbiota, as previously suggested [18]. Moreover, the relatively enriched *Lactobacilli* in some “doing-the-month” milk may also help to maintain mammary health, as suggested previously [9,10,11], and this is worthy of further investigation. However, additional studies are needed to further dissect the genetic factors and the program of “doing-the-month” driving the above variation and association.

As mentioned above, in our previous study we reported an only about 21% prevalence of *Lactobacillus* species in breast milk from healthy mothers in Taiwan [32]. However, our current data revealed a much higher prevalence of *Lactobacilli* in breast milk. These new data also support that *Lactobacilli* can be easily detected in most human milk, but their abundance may vary. Our data supports the association of the “doing-the-month” program with a higher population of *Lactobacilli* in some breast milk samples. However, the effects of “doing-the-month” seem to be minor in the *Bifidobacterial* population here. Statistical analysis identified that the *Bifidobacterial*-related bacteria were enriched only in the milk-H group (“non-doing-the-month” milk). A possible reason for this may be the relatively minor population of *Bifidobacterial* bacteria in the two “doing-the-month” milk groups. For example, according to the relative abundance of each bacterial member between the milk groups, *Bifidobacterial* members account for approximately 0.07%, 0.03%, and below 0.01% in the milk-H, milk-PCA, and milk-PCB groups, respectively. Furthermore, the prevalence of *Bifidobacterial* bacteria in the milk-H, milk-PCA, and milk-PCB groups was 32.6%, 62.9%, and 57.1%, respectively. Therefore, although *Bifidobacteria* were enriched in milk-H, their incidence was two-fold higher in the “doing-the-month” milk groups than that in the “non-doing-the-month” milk group. As described above, all the postpartum mothers were randomly recruited from three institutes, and these findings suggest that the practice of “doing-the-month” may still play some role in the distribution of *Bifidobacteria*, although this warrants further investigation. Our data also shows that *Bifidobacteria* are a minor bacterial population regardless of milk group (below 1% relative abundance), but we propose that the presence of *Bifidobacteria* in 32.6% milk samples of milk-H, 62.9% milk samples of milk-PCA, and 57.1% milk samples of milk-PCB might still play a role in acting as a reservoir of *Bifidobacteria* for the infant’s gut microbiota. Previous studies have reported that the colonization, adherence, and survivability of potential probiotics in the gut tract is important, and even a small inoculum could have a great impact [43,44,45]. Moreover, infants could consume approximately 556 to 705 mL/d breast milk by 6 days after birth [46], and 15-week old infants could consume approximately 788 mL of milk per day [47]. Furthermore, the total bacterial counts in human milk from healthy mothers ranged from 40 to 710,000 CFU/mL [48]. Another report indicated that a baby could ingest about 800 mL of milk daily, consuming approximately 8 × 10^4^ to 8 × 10^6^ commensal bacteria while suckling [49]. In summary, the microbial content accumulated in the baby’s gut through daily breast-feeding could be quite significant, implying that the relatively low abundance but higher prevalence of *Bifidobacteria* in most “doing-the-month” milk groups could still play an important role in the infant gut. Furthermore, in comparison to a recent report in Taiwan, the current data also support a relatively lower abundance of *Bifidobacteria* in most milk samples in Taiwan [32]. Therefore, the factors that may lead to the relatively lower prevalence and abundance of *Bifidobacteria* in human milk in Taiwan should be further explored. We speculate that the minor amount of *Bifidobacteria* in the current milk samples (regardless of milk groups) might still play a role in acting as a source for the infant’s gut. On the other hand, several studies indicate that the major classes of bacterial metabolites, the short-chain fatty acids (SCFAs), exhibit prebiotic and bifidogenic effects [50,51,52], and these support that the milk-derived *Bifidobacteria* could be further boosted by human milk or SCFAs in the infant intestine.

In our previous study, we reported that *Bifidobacteria* are rare bacterial members in human milk from healthy mothers in Taiwan [32]. However, the current data reveal an abundance of *Bifidobacteria* (about 32% to 62% prevalence) in breast milk compared to our previous report. These new data suggest that *Bifidobacteria* could still be easily detected in current human milk, although the abundance may vary.

Our data proves that the “doing-the-month” milk could be associated with enriched *Lactobacillus* bacteria or the relatively higher prevalence of *Bifidobacteris* than “non-doing-the-month” milk. In recent reports, the human milk oligosaccharide (HMO) profile has been linked with the milk microbial profile in breast milk. For example, several in vitro models have revealed that *Bifidobacterium* and *Lactobacillus* are able to use specific HMOs for growth [53,54]. Moreover, it is known that HMO differs between secretor and non-secretor mothers [55,56], and differences in the microbiota composition and quantity in human milk have recently been found to be dependent on the secretor/non-secretor status during the first 4 weeks of lactation [57]. Notably, *Lactobacillus* spp., *Enterococcus* spp., and *Streptococcus* spp. were found to be lower in non-secretor samples than in secretor samples. *Bifidobacterium* genus and species are less prevalent in non-secretor samples [57]. These previous findings indicate that the secretor samples could harbor many more *Lactobacillus* and *Bifidobacterium* bacteria in milk. In the current study, we did not detect the HMO profile between the three milk groups, but all the milk samples here were randomly donated by postpartum mothers. Thus, we could not rule out the possibility that the relatively enriched *Lactobacilli* and the more prevalent *Bifidobacteria* in current “doing-the-month” milk could be partially attributed to HMO. Notably, as mentioned, a previous study indicated that HMO could be linked to both higher *Lactobacillus* and *Bifidobacterium* bacteria in milk [57]. However, in our current study, the “doing-the-month” milk-PCB sample was associated with much higher *Lactobacillus* bacteria but less prevalent *Bifidobacteria*. Collectively, we speculate that the practice of “doing-the-month” could play a much more important role than HMO in the milk microbiota. In fact, other factors have been shown to influence the *Bifidobacteria* population in milk. For example, the total bacterial concentration of *Bifidobacterium* spp. was found to be lower in colostrum than in transitional or mature milk in a previous report. Moreover, *Bifidobacterium* was also reported to be higher in milk from mothers who reached full term, and vaginal births were associated with higher concentrations of *Bifidobacterium* as well [58]. In the current study, most milk donors in two “doing-the-month” milk groups gave birth through virginal delivery, and most milk samples were contained mature milk (day 16 and beyond). However, the *Bifidobacterium* concentration was still low in most milk samples. Thus, we could not find a relationship between vaginal births, lactation periods, and *Bifidobacterium* levels in the current milk samples.

In our study, we performed three alpha diversity analyses (Chao1, Shannon, and Simpson Reciprocal Index) to determine the microbial diversity between the three milk groups. The Chao1 index indicated that the practice of “doing-the-month” could be associated with a higher number of bacterial species in most milk samples of these groups (milk-PCA and milk-PCB). However, the Shannon and Simpson Reciprocal Index revealed that most milk samples could still display a wide profile of microbial diversity regardless of milk groups. Therefore, we used the beta diversity analysis (PCA, the weighted and unweighted variants of UniFrac distance analysis) to compare the overall differences in each milk microbial composition between the milk groups. According to the findings of the PCA analysis (beta diversity analysis), approximately 70% of the “doing-the-month” milk samples displayed a different or unique bacterial profile. In contrast, about 59% of the “non-doing-the-month” milk samples displayed a similar bacterial composition. Furthermore, the weighted (quantitative) variants of the UniFrac distance analysis also revealed that 56.4% of the milk-H samples shared two similar patterns (two clusters) of the bacterial profile. Moreover, this analysis also indicated that approximately 67% to 86% of the “doing-the-month” milk samples shared a different bacterial profile. Finally, the findings obtained by using the unweighted UniFrac analysis were in line with the results obtained by the PCA and weighted UniFrac metric distance analyses. Collectively, the beta diversity analyses demonstrated that a large portion of the samples in the milk-H group displayed a similar bacterial profile, but most milk samples of the “doing-the-month” milk group displayed a different or unique bacterial profile. This evidence implies that the practice of “doing-the-month” could lead to elevated microbial diversity in each milk sample (beta diversity analysis). In fact, “doing-the-month” programs in postpartum care centers have been associated with fewer physical and depressive symptoms among postpartum women [59]. Furthermore, a recent study investigated the mother’s postnatal psychosocial distress and variation in the milk microbiota and suggested a potential relationship between maternal psychosocial distress and the milk microbiota. This study indicated that the healthy group was linked with an increase in the diversity of the milk bacterial community, observed during the first 3 months of breastfeeding. This report suggested that the general increase in diversity could be explained by an increase in *Lactobacillus* and other minor genera, together with a decrease in *Staphylococcus*. Moreover, progressive and distinct changes in the levels of *Firmicutes*, *Proteobacteria*, and *Bacteroidetes* at the phylum level and *Acinetobacter*, *Flavobacterium*, and *Lactobacillus* at the genus level were also found in the milk samples of women with low psychosocial distress [60]. Notably, this previous report also revealed that no significant differences in the relative abundance of major bacterial genera were detected between women with high and low psychosocial distress [60]. Our data here support the previous report that the “doing-the-month” may not affect the distribution of major bacterial genera in milk in that *Staphylococcus* and *Streptococcus* are still the more abundant bacterial genera regardless of “doing-the-month” and “non-doing-the-month” milk samples. Furthermore, our data also provide clues that the practice of “doing-the-month” indeed plays a role in the relative minor bacterial population in milk, and is linked with relatively prevalent or abundant *Lactobacilli*, *Bifidobacteria*, or *Archaea* in milk. However, whether our findings were driven by the fewer physical and depressive symptoms in postpartum women should be further investigated.

Next, we would like to discuss the potential roles of “doing-the-month” on other bacterial members. LEfSe analysis identified 33, 20, and 8 groups of bacterial genera enriched in the milk-H, milk-PCA, and milk-PCB groups, respectively (Figure 2). Intriguingly, among the identified bacterial members, the “non-doing-the-month” milk contained more common skin-, oral, and enteric-related bacterial profiles. For example, among the identified bacterial family or genus enriched in milk-H (“non-doing-the-month” milk), *Gemella*, *Enterococcus*, *Pseudomonas*, *Bifidobactericeae*, and *Bifidobacterium* have often been detected in human milk samples, and are typically identified as the skin-, enteric-, or oral-associated genera [61,62,63]. Moreover, the family_X1, *Pseudomonadaceae*, *Neisseriaceae*, *Alteromonadales*, and *Paenibacillaceae* have often been detected in human milk samples [64,65]. In contrast, the milk samples from milk-PCA and milk-PCB groups were found to be enriched with rare (less detected) or uncommon bacterial members in breast milk. For examples, the archaea-related bacterial members are enriched bacterial members in both of the two “doing-the-month” milk groups. Intriguingly, some recent reports have also detected the presence of Archaea in human milk or colostrum samples [19]. At present, the roles of those archaea in human milk are not well understood, and possibly some of them could be involved in weight regulation [66,67,68]. However, all these factors need to be further investigated, and it is of interest to further study the roles of Archaea in human milk and their transfer from human milk to the gut of breast-fed infants in our next study.

Next, we would like to compare the major bacterial population between the “doing-the-month” and “non-doing-the-month” milk groups. First, referring to the major bacterial member between the milk groups, in agreement with previous reports [19], our findings also support that the most common and abundant bacteria are *Staphylococcus* and *Streptococcus*, although different proportions have been recognized regardless of milk groups. In other words, environmental-, skin-, enteric-, or oral-related bacteria are universal in human milk [19]. However, different proportions of specific bacterial members have been observed between the three milk groups here. For example, the milk-H group contained more *Rothia* (6.2%) and *Enhydrobacterv* (5%), while milk-PCA contained more *Corynebacterium* (6.6%), and milk samples from the milk-PCB group were dominated by *Acinetobacter* (28%) and *Lactobacillus* (8.7%). In summary, our data indicate that the three milk groups could be associated with common and major bacterial members, but also harbor quite different and characteristic bacterial members. At present, the roles of these common bacterial members in milk are not well understood, but reports indicate that random bacterial isolates collected from breast milk samples of healthy lactating women could display anti-bacterial activities against the growth of *S. aureus* [49].

Although some findings are contradictory, some factors have been reported to affect the milk microbiota. For example, HMO, BMI, gestational age, delivery mode, intrapartum antibiotics, biological sex, parity, lactation stage, diet, composition of human milk, HIV infection, geographic location, collection method, and feeding method could affect the milk microbiota differently [19,57]. Our study demonstrates that the practice of “doing-the-month” could indeed influence the milk microbiota, and possibly all the procedures within that practice or the factors mentioned above could contribute partially to the milk microbial composition. It is worth noting that a recent study suggests that, in addition to the abundance of microbes, the interactions between these factors in milk are important [69]. Additionally, evidence also indicates that the breast milk microbiota could be affected by breast-feeding as well, in the retrograde inoculation, whereby the microbiota in the infant oral cavity also affects the milk microbiota [21].

The limitations of this study are that no analysis was undertaken to compare the relationship between the milk microbiota and the potential roles of BMI, delivery types, chemotherapy, mental status of postpartum mothers, HMO profile, dietary content, and the potential retrograde inoculation through microbiota colonized in the infant oral cavity. Moreover, it is not possible to further analyze the potential roles of the group practicing herbal therapies and the milk microbiota due to the limited sample size and the fact that some herbal medicine recipes are recognized as business secrets. Since this was a pilot study, all the mothers were randomly recruited in postpartum care centers, contributing to a diverse background of mothers. Furthermore, the other limitations are that the “doing-the-month” practice is not proposed to all women. Firstly, it should be indicated that a study reports that the “doing the month” is not necessarily protective and supportive of postpartum women [28], and the traditional practices such as activity restriction (the practices of inactivity) could be unhealthy for some postpartum women, both physically and psychologically [27]. Secondly, the herbal therapy or supplements in “doing-the-month” may not be suitable for all women, in that women may suffer adverse effects caused by common herbs [28]. Finally, it is necessary to pay money to practice “doing-the-month” in postpartum care centers because these centers are staffed by physicians and midwives who are responsible for caring for the baby and new mothers. Therefore, it is important to select a postpartum care center that could combine modern healthcare delivery practices with traditional ritual practices for new mothers [70], and these may help to balance the potential benefits and risks of the “doing-the-month” practice.

In conclusion, our data suggests that the practice of “doing-the-month” could be linked to a diverse range of milk microbial composition, and this practice could be associated with higher chances of prevalent *Bifidobacteria* and abundant *Lactobacilli* in milk samples. Finally, the “doing-the-month” is also relevant to higher chances of more bacterial species or Archaea in milk samples. The current pilot study has paved the way to determine the roles of “doing-the-month” on manipulating the milk microbiota, and provides new evidence that it could also benefit postpartum mothers with respect to their milk microbiota.

## Figures and Tables

**Table 1:** Information for the “doing-the-month” and “non-doing-the-month” milk groups. Milk samples were collected from postpartum mothers who had completed the practice of “doing-the-month” (postpartum caring) for 20 to 30 days in postpartum care center A (milk-PCA, *n* = 14) or B (milk-PCB, *n* = 27). The control milk samples were harvested from the postpartum mothers in hospital who did not conduct the “doing the month” (milk-H, *n* = 46).

Milk Origin/Group	Postpartum Care Center A (Milk-PCA)	Postpartum Care Center B (Milk-PCB)	Obstetrics Department (Milk-H)
Sample size	14	27	46
Age for mothers who enrolled in study, year	22 to 43	22 to 38	17 to 43
Duration for Mother stayed in care centers, day	≥20 (20 to 30)	≥20 (20 to 30)	0
Services in care centers			
Meal plan	Dietary therapy and herbal therapy ^a^	Dietary therapy and herbal therapy ^a^	None
Meal frequency	3 to 5 meals/day	5 meals/day	None
Content for dietary and herbal therapy	Seafood, Shi-Quan-Da-Bu-Tang (ten-significant tonic decoction) Ba-zhen-tang (eight-treasure decoction) Eucommia leaf tea, herbal medicine recipes (business confidentiality) Herbal tea (promote lactation)	Seafood Sesame oil chicken Sheng-hau-tang, Siwu-Tang Eucommia leaf tea herbal medicine recipes	

^a^ Based on traditional Chinese theory, the postpartum care center provides “warm” dishes and “warm” herbal therapies which were created and supervised by the doctors and dietitians in the center.

**Table 2:** The prevalence of *Lactobacillus* or *Bifidobacterial* bacteria in the three milk groups.

Type of Milk Group	Occurrence, % ^a^	
	*Lactobacillus Bacterium*	*Bifidobacterial Bacterium*
Milk-H ^b^ (n = 46)	86.9	32.6
Milk-PCA ^b^ (n = 14)	100	62.9
Milk-PCB ^b^ (n = 27)	92.8	57.1

^a^ Calculated by the numbers of milk samples that had been detected with lactobacilli or bifidobacteria divided by the total milk samples in the same milk group. ^b^ Milk samples were collected from postpartum mothers who had completed the practice of “doing-the-month” (postpartum caring) for 20 to 30 days in postpartum care center A (milk-PCA, *n* = 14) or B (milk-PCB, *n* = 27). The control milk samples were harvested from the postpartum mothers in hospital who did not conduct the “doing the month” (milk-H, *n* = 46). The two postpartum care centers provided different dietary and herbal therapies, as shown in Table 1.
